# Strategies and Challenges for Successful Implementation of Green Economy Concept: Edible Materials for Meat Products Packaging

**DOI:** 10.3390/foods10123035

**Published:** 2021-12-07

**Authors:** Roxana Gheorghita Puscaselu, Liliana Anchidin-Norocel, Ancuţa Petraru, Florin Ursachi

**Affiliations:** 1 Faculty of Medicine and Biological Sciences, Stefan cel Mare University of Suceava, 720229 Suceava, Romania; roxana.puscaselu@usm.ro; 2 Integrated Center for Research, Development and Innovation in Advanced Materials, Nanotechnologies and Distributed Systems for Fabrication and Control, Stefan cel Mare University of Suceava, 720229 Suceava, Romania; 3 Faculty of Food Engineering, Stefan cel Mare University of Suceava, 720229 Suceava, Romania; ancuta.petraru@fia.usv.ro (A.P.); florin.ursachi@fia.usv.ro (F.U.)

**Keywords:** agar, sodium alginate, glycerol, environment, salami

## Abstract

Currently, the problem of pollution due to plastic waste is a major one. The food industry, and especially that of meat and meat products, is intensely polluting, both due to the raw materials used and also to the packaging materials. The aim of the present study was to develop, test, and characterize the biopolymeric materials with applications in the meat industry. To obtain natural materials which are completely edible and biodegradable, different compositions of agar, sodium alginate, water and glycerol were used, thus obtaining 15 films. The films were tested to identify physical properties such as smell, taste, film uniformity and regularity of edges, microstructure, color, transmittance, and opacity. These determinations were supplemented by the evaluation of mechanical properties and solubility. According to the results obtained and the statistical interpretations, three films with the best results were used for packing the slices of dried raw salami. The salami was tested periodically for three months of maintenance in refrigeration conditions, and the results indicate the possibility of substituting conventional materials with the biopolymer ones obtained in the study.

## 1. Introduction

All over the world, the consumption of meat products is high due to the accessibility of such products, but also to the varied range available to the consumer. Unlike other foods, ready-to-eat sliced meat products, such as salami or sausages, are more susceptible to external contamination and have a short shelf life. In the case of these products, it is very important to obtain a hygienic product, therefore, during the entire obtaining process, the pre-established conditions must be observed. A good packaging material must prevent the transfer of constituents from packaging to product and prevent distortion of nutritional and sensory qualities [1]. Throughout the production process, from manufacturing to the final consumer, pasteurization, cold-drying and packaging are very important [2]. Unfortunately, the meat industry is intensely polluting throughout all stages of the technological process [3]. An important step in maintaining the nutritional and sensory qualities is the used packaging. It is important to choose a material that preserves the properties of the packaged product and ensures the safety of its ingestion. Meat products facilitate the development and proliferation of microorganisms due to the high content of water activity and nutrient availability [4,5]. According to studies, the main reason for the proliferation of sporulation and the reduction of the shelf life is the choice of inappropriate packaging material or improper packaging conditions [6]. Unfortunately, at this time, most of the packaging materials used are heavily polluting, plastic-based, obtained from non-renewable, oil-based resources. Also, due to their complex character, being usually composed of several types and layers of material, they are difficult to sort and recycle, representing a real problem of environmental pollution. Globally, authorities are looking to identify new strategies to reduce pollution and use biodegradable and compostable materials. Since 2004, the European Union has also established Regulation No 1935/2004, with regulations that refer to all materials that come into contact with food [7]. These have been updated and, in 2008, enhanced with new regulations that refer to the conditions that should be met by all materials that come into contact with food products [8,9]. The European Union’s plastic strategy, which includes rethinking the use of plastic in a more sustainable way and replacing multilayer materials, comes into effect in 2030 [10]. Until then, research should bring to the fore strong reasons for the use of other substances to the detriment of plastics, and European programs such as Horizon Europe 2020 encourage these actions [11]. Lately, biopolymers obtained from algae have attracted the interest of researchers, manufacturers, and consumers, mainly due to its low cost and ease of reproducibility. Biopolymeric materials are non-toxic, non-allergenic, biodegradable and, depending on the production process, even edible [12]. Unfortunately, due to the natural character and the composition that can be influenced depending on the climatic conditions and the production area, the manufacturing technologies would be constantly improve, in order to obtain a product with similar characteristics every time [13]. This can represent an inconvenience of natural materials as opposed to the use of plastic which, due to its synthetic character, can always be reproduced in the same conditions when the manufacturer follows the same instructions for development. Even so, the industry has constantly researched and identified new solutions for the development of materials with characteristics similar to conventional plastic that are very harmful to the environment. In this sense, new compositions were identified, the synergistic nature of some biopolymers and the possibility of adding plasticizers or natural substances such as powders or essential oils were observed, and the already existing characteristics were improved [14,15,16,17,18,19]. According to studies and literature, biopolymer films have been tested as packaging materials for a wide range of foods: dairy products [20,21,22,23], sweets [24,25], powdered products [26], food supplements [27,28] and others. When testing the possibility of replacing conventional packaging with biopolymeric ones for meat and meat products, the results obtained indicated improved physicochemical and sensory properties, prolonged their shelf life, and prevented the growth of microorganisms, with the real possibility of replacing conventional packaging with the newly tested ones [29,30,31,32]. 

The present study aimed at developing a packaging material based on agar and sodium alginate, and plasticized with glycerol. Agar is an intense biopolymer used to obtain biodegradable materials due to its good film-forming properties and high efficiency to form blend materials [33]. However, they depend on the type of agar, its origin or the method of obtaining and are known as transparent and heat-sealable, but with higher retraction ratios than other biopolymers [34]. Sodium alginate, although initially used in the food industry, has expanded its applicability due to its properties: good mechanical strength, moisture barrier and cohesiveness. Other characteristics such as high water viscosity, permeability, absorption capacity, ability to incorporate various compounds into the matrix, and its wound-healing properties have extended its applicability in biomedicine and in the development of new materials [35]. Glycerol, unlike other plasticizers due to its small molecules, can be easily inserted into the free areas of the biopolymer matrix, thus obtaining a much more compact structure. When used in the development of biopolymer films, it helps to obtain more flexible, softer materials and reduce stiffness, but it increases water permeability [36].

After obtaining the films, they were developed and tested, and three of them were used as packaging material for slices of raw-dried salami. The food product packaged in biopolymeric material was tested for three months, and the results indicate the ability of these foils to be used as packaging material. The study is one of interest because, from our searches in the literature, to this point no tests have been made to target the packaging with biopolymers and the edible materials of raw-dried and smoked salami. Thus, according to the results, the films developed and tested in this study can be used as packaging materials for meat products and can replace the conventional ones. The results are also reinforced by those in the literature when biopolymer materials applied to other meat and fish products have improved the sensory, nutritional and microbiological characteristics of packaged food. This can represent a basis for fundamental and industrial research and application.

## 2. Materials and Methods

### 2.1. Materials

In order to develop new materials for food packaging, biopolymers such as agar and sodium alginate were used. Glycerol was used to plasticize the material. All products were purchased from the company Sigma Aldrich, the Romanian subsidiary.

### 2.2. Development of Biopolymers—Based Films

In order to develop the new biopolymeric materials, 4.5 g of the mixture agar (0.5–3 g), sodium alginate (0.5–3 g), glycerol (0.5–1 g) and 150 mL of distilled water was used, according to the Table 1. The composition used facilitated the obtaining of films with a size of 30 cm × 60 cm.

The films were obtained by the casting method, according to the method used by Gheorghita et al. [37] and the film-forming solutions were kept on silicone support until complete drying (38–72 h, ambiental temperature, 23 ± 2 °C, rH 54 ± 2%). After obtaining, they were tested in order to identify the films with characteristics appropriate for the meat industry.

### 2.3. Evaluation of Physical and Optical Properties

The evaluation of the physical properties involved the observation of the samples immediately after obtaining, and then following the uniformity of the films, monitoring the presence of pores and fissures and the regularity of the edges, and the presence of any insoluble substances or defects on the surface or structure of the films.

The thickness of the films was tested using a digital micrometer with an accuracy of 0.001 mm (Mitutoyo, Kawasaki, Japan), and the final result was noted after at least 10 readings made on different areas on the entire surface of the films. To identify the retraction ratio, the thickness of the film-forming solution and the final thickness of the films were taken into account, and the results were noted in the formula below:

$$\mathrm{Retraction}\;\mathrm{ratio}\left(\%\right)=\frac{T1-T2}{T1}*100$$

where T1—thickness of the film-forming solution (μm) and T2—thickness of the film (μm).

The microstructure of the films was visualized using the Mahr CWM 100 confocal microscope (MarSurf, Göttingen, Germany) by observing the entire surface of all the films. After obtaining the microtopographs of the films, their uniformity, the presence of pores or fissures undetectable at first sight, the regularity of the edges, as well as any incomplete solubilized or insolubilized substances were observed. For the identification and measurement of roughness, but also of other defects, the trial version of the version 9 of Mountains^®^ software for Surface Imaging, Analysis and Metrology Software (Digital Surf, Lavoisier, France) was used.

In order to identify moisture content (MC), 3 cm × 3 cm specimens were initially weighed and reweighed after being maintained for 24 h at a temperature of 110 °C. The hot air oven, without ventilation, was used for drying (Memmert, Germany). After performing the determinations in triplicate, the formula was applied:

$$\mathrm{MC}\;\left(\%\right)=\frac{W0-W1}{W0}*100$$

where W0—mass of the initial sample (g) and W1—mass of the sample after drying, (g).

The color of the films was identified using the Konika Minolta CR-400 colorimeter, and the final results of the three parameters—L*, a*, b* were noted after at least ten readings made in different areas of the film surface. To evaluate the transmittance (Tr) and opacity (O), specimens the size of a spectrophotometer tank (1 cm × 3 cm) were tested by reading the corresponding absorbance at some well-established wavelengths using the Ocean Optics HR 4000 CG-UV-NIR (Ocean Optics, 830 Douglas Ave., Dunedin, FL, USA) spectrophotometer. Thus, to identify the transmittance for each sample, the absorbance was read at a wavelength of 660 nm, and the determinations were made in triplicate. The opacity was calculated using the formula below, taking into account the absorbance read at the wavelength of 600 nm:

$$\mathrm{Opacity}\;\left(A*\mathrm{mm}-1\right)=\frac{A}{T}$$

where A—absorbance read at specific wavelength, and T—thickness (mm).

### 2.4. Determination of Mechanical Properties

In order to identify the mechanical performances, 1 cm × 10 cm film specimens were tested according to STAS ASTM D882 (Standard Test Method for Tensile Properties of Thin Plastic Sheeting) [38]. For testing, the universal texturometer ESM Mark-10 loaded with a 5 kN cell was used. For evaluation, grips specially designed for testing thin films and foils were attached. The travel speed was set at 10 mm/min, and the working temperature stabilized at 26 ± 0.4 °C. Tensile strength (TS) was calculated according to formula:

$$\mathrm{TS}\;\left(\mathrm{Mpa}\right)=\frac{F}{S}$$

where F—maximum applied force (kN), and S—area (mm^2^).

The elongation (E) was calculated with the formula, where Δl represents the distance between the final and the initial length l, (mm), and represents the capacity of the material to expand until the rupture occurs:

$$E\;\left(\%\right)=\frac{\Delta l}{l}*100$$

In order to establish a more accurate result, the mechanical testing of the samples was performed in triplicate.

### 2.5. Evaluation of Solubility Characteristics

For identifying the stability of the material at the external storage conditions (humidity, temperature), but also its resistance depending on the packaged product, the testing of the solubility characteristics was taken into account by measuring hydration capacity and water solubility. These were supplemented by the determination of the water activity index.

For the rehydration capacity (WS) testing, specimens similar to those used for moisture content testing (3 cm × 3 cm) were weighed and immersed in a Duran tube with 50 mL of distilled water and maintained for 8 h. After 8 h, the films were removed from the liquid, lightly taped with filter paper and placed in a hot air oven being maintained for 24 h at 110 °C for drying. Subsequently, they were re-weighed, and the values obtained were entered in the formula:

$$\mathrm{WS}\;\left(\%\right)=\frac{W0-W1}{W0}*100$$

where W0—the mass of the sample before immersion in water, (g), and W1—the mass of the sample after the 8 h, (g). 

The swelling ratio (SR) was determined by using 3 cm × 3 cm specimens immersed in 50 mL of water, for a period of time ranging from 30 s to 20 min. The film samples were weighed before and after immersion in water, and the values were used in the following formula, where Wt—the mass of the sample after holding in water for a time *t*, (g), and W0—the initial mass of the sample, (g):

$$\mathrm{SR}\;\left(\%\right)=\frac{\mathrm{Wt}-W0}{W0}*100$$

Water solubility determination was performed in triplicate and swelling ratio evaluation in a single experiment.

The water activity index (a_w_) was determined using AquaLab 4TE equipment (Meter Group, Munchen, Germany), and the results were established after at least 5 readings in different areas of the film surface. The determinations were performed at temperatures of 25 ± 0.41 °C.

### 2.6. Identification of Microbiological Stability

To identify the incidence of microorganisms on the surface of the films, plates with dehydrated culture media (NISSUI Pharma), specific for total count (TC), *Escherichia coli* (EC), coliforms (CF), *Staphylococcus aureus* (XSA) were used. *Listeria monocytogenes* (LM), enterobacterias (ETB), yeasts and molds (YM) were useful. For this purpose, 1 g of film was homogenized with 9 mL of microbiologically inert solution. After strong homogenization, 1 mL was used to hydrate the culture media. The plates were thermostated at 37 °C for 24–48 h to identify bacteria strains and 72 h for yeasts and molds.

### 2.7. Testing of Food Packaged in Biopolymer Foils

The slices of dried raw salami were purchased from a national Romanian producer. 100 g of product was obtained from 138 g of pork of EU origin, salt, dextrose, spices and flavors. The product is a smoked type, with natural smoke obtained from hardwood. According to the manufacturer, the average nutritional value per 100 g of product is: 37 g fat, 0.6 g carbohydrates, 23 g protein, and 3.5 g of salt. The energy value is 1770 kJ/427 kcal per 100 g of product. The product is sold sliced, with 30 pieces per package, and has a shelf life of 90 days according to the specifications offered by the manufacturer. They were kept in a dry and cool place (refrigeration temperatures, 4 °C ± 1 °C) and consumed within a maximum of three days of opening. To identify the possibility of using films as packaging materials for salami slices, only the films with the best results were chosen—S4, S8 and S13. The salami slices were packed in these foils and were glued by thermal welding at 170 °C, for 5 s. Packages were made with individual slices of salami, which were kept in cardboard boxes and analyzed at different times, such as one week, one month, two and three months after packaging. For comparison, the slices packed in biopolymer foils were characterized in relation to those kept in the original package. During the test period, the changes in the mass of the product, the color parameters (L*, a*, b*), the water activity index and its acidity were verified. The color variations between the initial moment and the subsequent test periods were determined using the following calculation formula:

$$\Delta E=\sqrt{{\left({\Delta L}^{*}\right)}^{2}+{\left({\Delta a}^{*}\right)}^{2}+{\left({\Delta b}^{*}\right)}^{2}}$$

where ΔE represents the total color differences, and ΔL*, Δa*, and Δb* are the differentials between the sample color parameter after tested period and the color parameter from the initial moment.

Variations in the mass of packaged samples were checked by weighing the salami slices at analytical balance and expressed in grams. The color and water activity index were evaluated with the same colorimeter and water activity equipment as the biopolymer films, respectively. 

To determine the acidity, neutralization of the free acidity with 0.1 N sodium hydroxide solution was tested in the presence of phenolphthalein as an indicator. Thus, 1 g of fat was placed in a 100 mL Erlenmeyer flask, and the vessel was heated on a water bath until the fat was completely melted. 20 mL of the alcohol-ether mixture and a few drops of phenolphthalein were added and titrated with sodium hydroxide solution until a pale pink color was reached, and persisted for 30 s. Acidity is expressed as a percentage of oleic acid.

All determinations were performed in triplicate.

### 2.8. Statistical Analysis

Statistical analysis was performed with XL Stat 2019 software, and the optimization with Design Expert 12 (Stat Ease, Godward St NE, Suite, Minneapolis, MN, USA). Data analysis was assessed using the Minitab Statistical Software (Coventry, UK).

## 3. Results

### 3.1. Evaluation of the Developed Biopolymer-Based Films 

Immediately after obtaining, the films were tested from the sensory point of view, but also from the physical properties or from the oral solubility. The results are noted in Table 2.

According to the Table 1 and Table 2, films with higher amount of agar and low of glycerol into composition presented low solubility, and were rough on the outside and with low solubility—**S7**, **S9**. Increasing the content of sodium alginate or plasticizer mass has led to glossier, finer, pleasant to the touch films, but has had an effect in increasing adhesion and reducing oral solubility—**S4**, **S8**, **S13**. The same results were obtained by Singh et al. [39] when using sodium alginate for green packaging material development. The higher glycerol content facilitated the obtaining of films with a sweeter taste that was easily perceptible but pleasant—**S3**, **S4**, **S8**.

**Figure 1:**
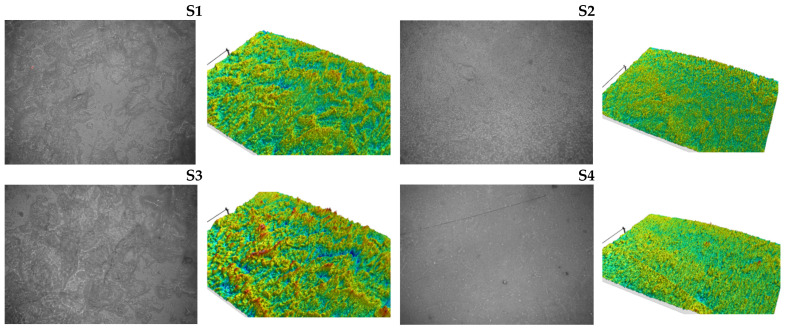

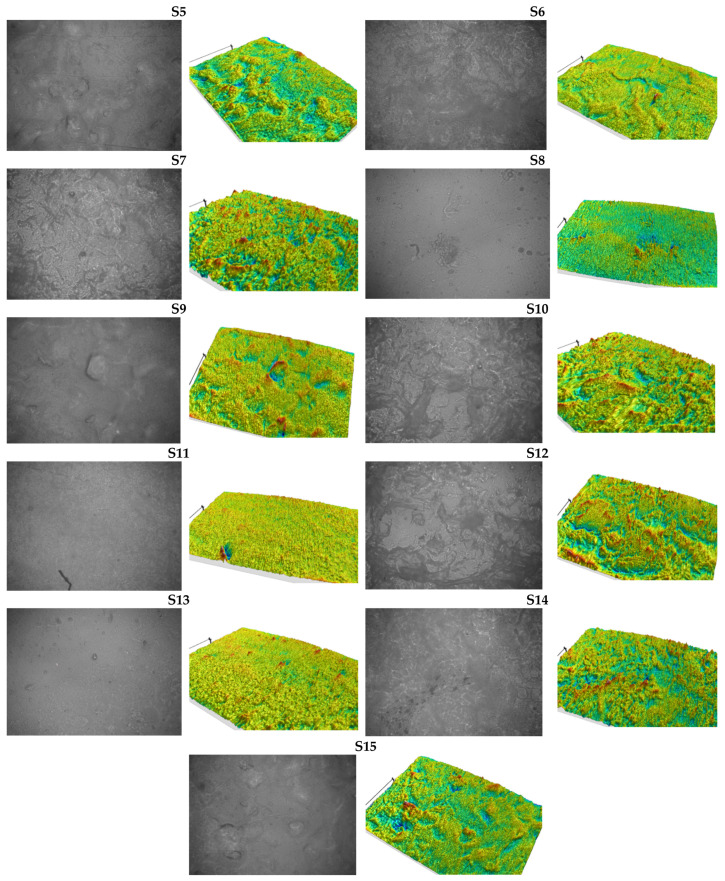
Microscope images and microtopographies of films.

The microstructure of the foils can be observed in Figure 1. According to SEM images and microtopographies, the homogeneity is greatly influenced by the films’ composition. Thus, the decrease of the plasticizer content had an effect in obtaining films with a less smooth microstructure—**S11**–**S15**, and with higher fragility and brittleness. The results obtained are not surprising, given that they were also found in other research studies that have focused on the importance of plasticizers and, implicitly, glycerol, on the development of biopolymer films. Thus, when added to the film-forming composition, the plasticizer improved the physical and mechanical properties [40,41,42], but increased the solubility of the films obtained [43,44]. According to the results, the increase in glycerol concentration from 10 to 50% was directly proportional to the increase in water solubility (from 23.21% to 33.37%). In our study, the higher amount of glycerol used was 1.00 g and 22.22%, respectively. The amount was sufficient for the development of a film with superior physical and mechanical characteristics (**S4**).

The characteristics of the films developed, such as thickness, retraction ratio, transmittance, opacity, tensile strength and elongation are presented in Table 3, where it can be seen that the most special properties are in the case of **S4**, **S8**, and **S13** samples. For a thickness about 40 µm, it obtained higher values of transmittance (around 70%), the smallest values of opacity (2.95–3.80 A*mm^−1^), and higher retraction ratio values (more than 42.40%).

According to Table 1 and Table 3, the thickness was influenced by the mass of the agar—**S1**, **S3**, **S9**, **S14**. The increase in sodium alginate content (**S5**, **S8**, **S10**, **S11**, **S12**) facilitated the development of thinner films, with a higher retraction ratio, but with better optical properties (higher transmittance, lower opacity). The best results obtained after a tensile strength test was for **S8** film, followed by **S11** and **S15**, films with a high sodium alginate content in the composition. 

**S8** has high mechanical performance compared to other samples obtained with high alginate and sodium content in the composition [45]. The addition of agar improves the mechanical properties of biopolymer films, especially tensile strength, as observed by Harnkarnsujarit & Li [46], when, after the addition of agar, the obtained films showed higher strength and elongation, but also more compact microstructure.

The best elongation values are observed in samples with high sodium alginate content in the composition—**S2**, **S11**, **S15**. The same characteristics have been identified in other works that have followed the development of films based on biopolymers [47].

The color of the films (Table 4) was influenced by their composition. Thus, the samples with higher agar addition in the composition (**S4**, **S9**, **S13**) showed higher values of luminosity and parameter a*, and, implicitly, lower values of parameter b*.

Regardless of the composition used, all the films tested had a better transparency than other films obtained from sodium agar and alginate, with or without the addition of carrageenan in the composition [48].

**Figure 2:**
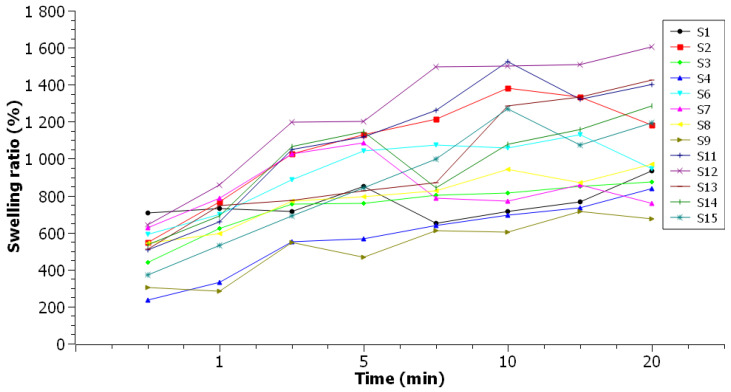
Swelling ratio testing.

The swelling ratio (Figure 2—representation of the average results of the testing) couldn’t be applied to the films **S5** and **S10** due to their very high solubility, as they did not retain their integrity even after 30 s of immersion. Films with high agar content in the composition presented low values of swelling ratio—**S3**, **S7**, **S9**, **S11** and **S12** films, with higher sodium alginate content in the composition showing high hydration capacity. However, the **S13** and **S14** samples deviate from the obtained results, which, although they have a high sodium alginate content in the composition, had high values of swelling ratio—1423.75% and 1286.89%, respectively. 

Statistical analyses (Principal component analysis, Pearson correlation and optimization) were used for identification of best characteristics of the films and then the utilization of these materials in food packaging.

The Pearson correlation (Table 5) showed a negative relationship between agar mass and water solubility (−0.715), thickness and retraction ratio (−1.000) and transmittance and opacity (−0.841). A positive correlation has been found between the transmittance and retraction ratio and thickness (both 0.698), alginate and water solubility (0.618) and also between glycerol mass and moisture content (0.561).

**Figure 3:**
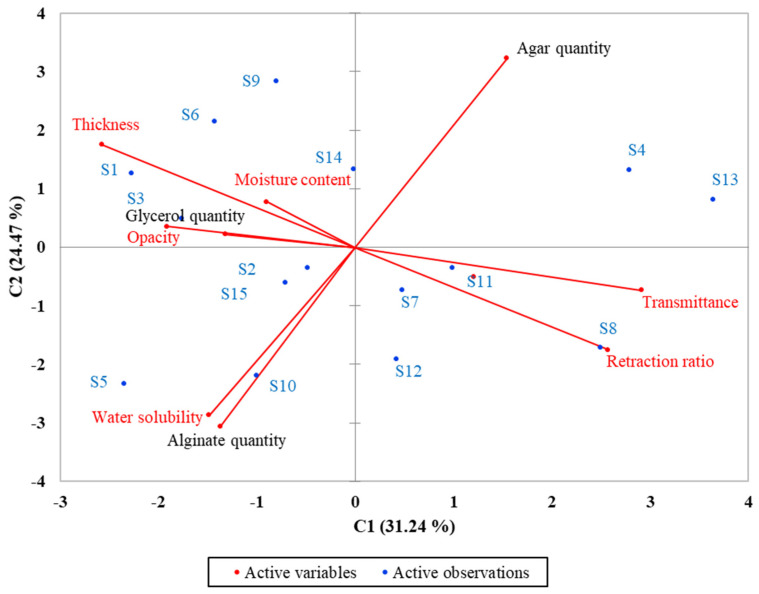
Principal component analysis (PCA) of the dataset consisting of analyzed.

Principal component analysis (PCA) was performed for all 15 samples (**S1**–**S15**) to highlight the correlation between quantities of some products (agar, alginate and glycerol) and the characteristics of the obtained films (thickness, moisture content, transmittance, retraction ratio, water solubility and opacity), and the results are presented in the biplot from Figure 3.

The PCA method limited data into two main components covering 55.71% of the variability (C1—24.47% and C2—31.24%). Analyzed parameters such as opacity, moisture content, and thickness were grouped around the glycerol quantity corresponding to **S1**, **S3**. Alginate content is positively correlated (samples **S5**, **S10**, **S12**) with water solubility, while agar content is negative correlated (**S13**, **S4**). Thickness has a significant negative correlation with retraction ratio (at the 0.01 level) for **S8** film and transmittance (at the 0.05 level) for **S11** film. **S2** samples has almost medium values and is located in the center of the biplot which proves that is transparent, without pores or fissures, well-defined edges, low solubility, allows multiple bending, and is flexible.

After microbiological testing, none of the microorganisms tested developed on culture media. These results can be reinforced by the very low value of the water activity index (below 0.4, Table 3). According to the results, the edible material is safe to ingest microbiologically. The substances used to obtain them are GRAS (Generally Recognized as Safe) and can be consumed in *quantum statis* doses. According to these characteristics, the materials obtained are of major interest, being useful both to the industry, but especially to the environment, their use generating zero waste.

### 3.2. Salami Packaged in Biopolymer Foils

Due to their better characteristics (microstructure without pores and fissures, color, and) mechanical properties, the **S4**, **S8** and **S13** films were chosen for use as packaging materials for the slices of raw-dried salami. Initially, **S14** foil was chosen as well, but it could not be used for packaging, as heat sealing could not be performed.

**Figure 4:**
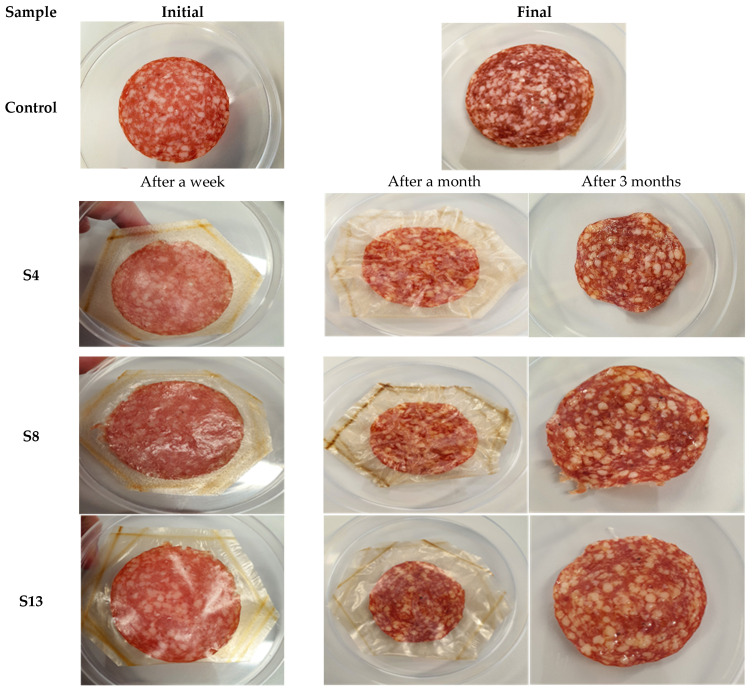
Meat products packed in biopolymers-based foils.

The images with the salami slices packaged and stored in such materials are presented in Figure 4.

As can be seen form Figure 4, the appearance of the slice of salami packed in **S13** foil looks like the one packed in the classic, multilayer package, obtained from successive layers of polyethylene, ethylene, metal foil and glue. The slices of salami packaged in foils **S4** and **S8**, although they had a lower mass (the packaging allowed the transfer of moisture content and, implicitly, the elimination of water from the product), still look very good. These confirm other results obtained by us when, by using agar, alginate, glycerol, and stevia, we developed a film with applications for the cheese and meat products industry. Thus, the prosciutto packaged in these biopolymeric materials and maintained for five months, kept its physical and microbiological characteristics better than the classic packaging [37].

The results obtained from the microbiological evaluation are presented in Table 6. If at the time initiated, immediately after packaging, none of the microorganisms tested did not develop on the culture media, after three months the presence of bacteria, yeasts and molds were identified.

According to the results, the salami slices packed in biopolymeric materials showed much higher microbiological stability. The results are of great interest because they can be used for the development of high-quality products, safe for consumption. The method can also be used to reduce the content of additives used with role in maintaining microbiological stability. The total number of germs is significantly lower in salami packed in polymer foils than in those distributed in conventional packaging. Rosario Morena et al. [49] highlighted the benefits of packaging salami slices in biopolymer films based on chitosan, but the microbiological load, after five days, although lower than the samples packed in conventional materials, was higher than that presented in this study (6 CFU/g for yeasts and molds and 5 CFU/g for bacteria in contrast to our results, when no yeasts and molds were developed on the culture media and the total count was 1 and 3, respectively, 15 CFU/g after three months of storage).

**Figure 5:**
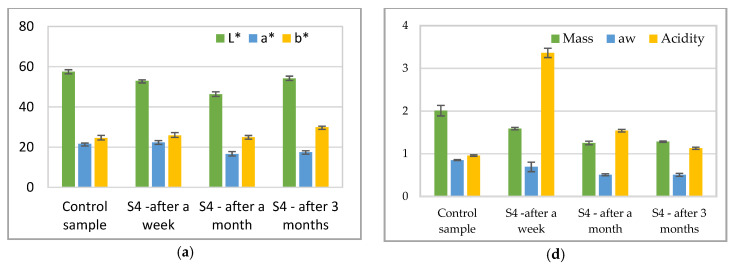

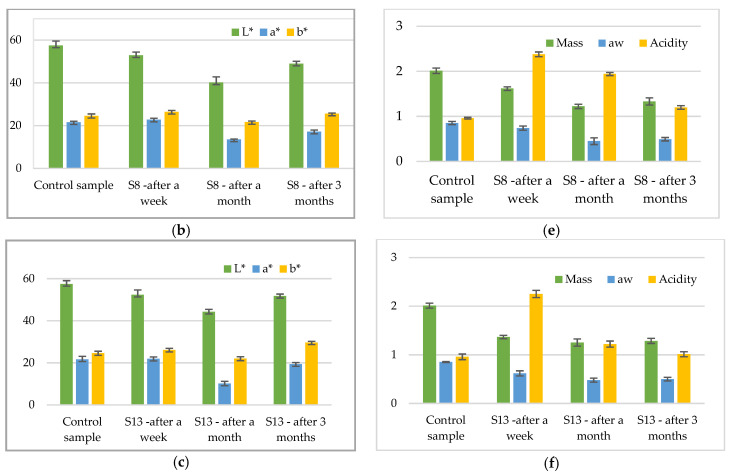
Physical-chemical parameters of sliced salami: Color parameters results for (**a**) salami slices kept in **S4** packaging material (**b**) salami slices kept in **S8** packaging material, (**c**) salami slices kept in **S13** packaging material; mass, a_w_ and acidity: (**d**) salami slices kept in **S4** packaging material (**e**) salami slices kept in **S8** packaging material, (**f**) salami slices kept in **S13** packaging material.

Results of physico-chemical parameters of meat samples are presented in Figure 5. The color of the samples analyzed showed a minor change after a week, and a major change after a month, but the differences between the three films are not so high for a* and b*, but more so for L*. For all samples tested, the luminosity was higher, which may influence lipid oxidation. Similar results were obtained by Lourenco et al., when packed lamb meat in foil based on sodium alginate [50]. Different results were obtained by Alexandre et al., when they packaged beef in sodium alginate and basil films. According to the study [51], the luminosity values were lower for the sample packaged in biopolymers. The authors acknowledge the beneficial effects of adding basil to the film matrix. The same beneficial effect of natural extracts was shown by Hosseini et al. [52] when they packed chicken breast in polymer foils with the addition of essential oils of lemon and verbena or by Kang et al. [53], when they packed low-fat frankfurters in sodium alginate film. 

Other research that has shown the beneficial effects of biopolymers in packaging meat and meat products has used chitosan in the composition [31,54,55]. Unfortunately, for solubilization, it involves the use of an acid, usually a high concentration acetic acid. Thus, even if the beneficial effects are visible, the sensory and nutritional characteristics of the packaged products are modified.

The color differences between initial time and after one week, one month, and three-month packaging are presented in Table 7. To evaluate the color changes over time, the initial values of the parameters L*, a*, b*, respectively L* = 47.126 ± 2.278, a* = 18.542 ± 0.898, b* = 20.772 ± 1.01 will be taken into account.

According to the data in Table 7, the smallest variations of the color parameters are found in the sample packed in foil **S8**. However, a reduction of the variation of the color parameters after a month of maintenance in the biopolymer packaging can be noticed, regardless of the type of packaging. According to the results, the salami slices individually packed in biopolymer foils showed less color variations than the control sample packed in conventional, multilayer material. The results are all the more satisfactory as, regardless of the packaging used, all samples were kept in the same refrigeration conditions. In terms of maintaining the original color of the food, the biopolymeric material was much safer than the plastic-based one.

The mass and water activity of the samples had similar values for the all of the films after the three months. Acidity after a week of storage increase and then after a month, respectively 3 months decrease because of the samples which lose water. The acidity of the packaged samples increased directly in proportion to their storage time. According to the results shown in Figure 5, slices of salami packed in biopolymer foils were more susceptible to lipid oxidation than those packaged in conventional materials.

According to the data obtained, each packaging material based on biopolymers can be used to preserve and maintain the shelf life of salami. The information specified by the manufacturer on the product packaging indicates that the shelf life of raw-dried salami, sold in individual slices is three months from obtaining. According to the results in Figure 5, after the first month of storage, the film S13 used for packaging kept the shelf life of the product better than other foils. Although salami slices packed in biopolymeric foils had higher acidity after the first week, it decreased after the first month, and can be compared with the values of the control sample. In the third month, the results are even better. Thus, biopolymer packaging tends to better protect the sensory and nutritional qualities of the product at one to two months of storage.

**Figure 6:**
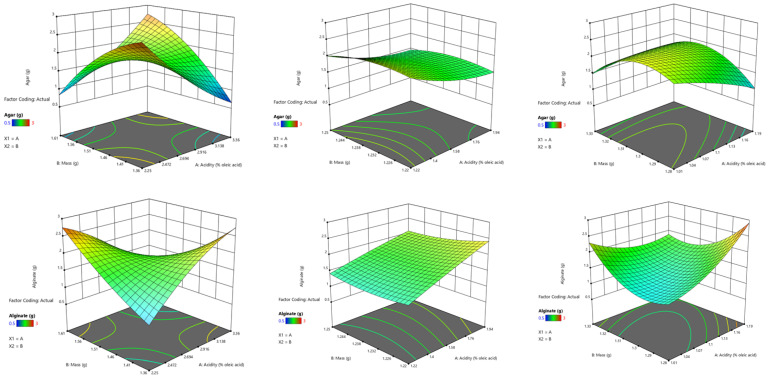

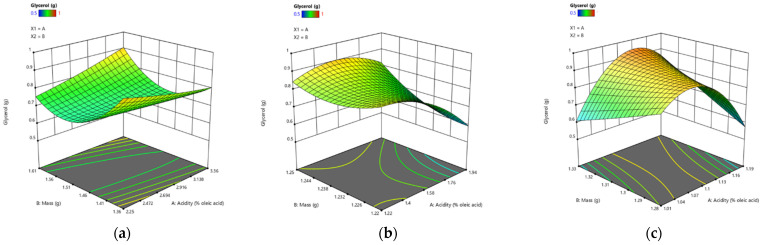
3 D Surface optimization of the films regarding acidity—mass of biopolymers/plasticizer (**a**) after a week, (**b**) after a month, (**c**) after 3 months.

Films optimization (Figure 6) was carried out for the three films with best characteristics (S4, S8, S13) which were used in meat packaging. Thus, the mass of biopolymers/plasticizer of films influenced the acidity of the meat in those three months and this aspect was determined by Design Expert 12 software. According to statistical data, acidity measured after a week was analyzed depending on the mass of biopolymers/plasticizer of films to observe which films are of a smaller value and the desirability obtained was: acidity 2.25, mass 1.61, glycerol 0.75, agar 0.83, alginate 2.75.

After a month, the desirability obtained was: acidity = 1.22, mass = 1.25, agar = 2.01, alginate = 1.41, glycerol = 0.84, and after three months the desirability was: acidity = 1.01, mass = 1.33, agar = 1.48, alginate = 2.30, glycerol = 0.61.

## 4. Conclusions

Environmental problems due to the use of plastic-based packaging, a non-renewable resource with unfavorable effects once in the environment, forced the identification of new compositions for the development of materials. The benefits of using biopolymers in the food industry were the reason for their choice for the development of new materials that were edible and biodegradable. Tests performed so far have identified biopolymers and compositions that can replace synthetic packaging.

The aim of the study was to develop new biobased-materials that can be used for food products packaging. The films based on biopolymers were obtained by the method of casting, from sodium agar and alginate, and plasticized with glycerol and water. After testing and identifying the best characteristics, three of the new films were used to package the slices of dried raw salami. The food samples were kept for three months in refrigeration conditions, according to the manufacturer’s instructions. The results obtained for foods packaged in biopolymer films are comparable to the control sample and the microbiological evaluation highlighted the safety of using these packaging materials to the detriment of conventional ones. The evaluation of the color differences showed that the samples packed in biopolymeric materials presented less color variations than the initial moment, regardless of the type of film used. However, sample mass and water activity were lower, with biopolymer membranes facilitating the transfer of moisture content more than conventional packaging. Microbiological analysis showed that the samples kept in the conventional packaging were more sensitive.

In addition to the benefits of testing, the use of such materials is extremely beneficial to the environment, as they are completely biodegradable, and generate zero waste. To improve the sensory, physico-chemical or microbiological properties, they may contain additions of other bioactive compounds.

## Figures and Tables

**Table 1:** Composition of the films.

Film	Agar, (g)	Sodium Alginate, (g)	Glycerol, (g)	Water, (mL)
S1	2	1.5	1	150
S2	1.5	2	1	
S3	1.75	1.75	1	
S4	3	0.5	1	
S5	0.5	3	1	
S6	1.875	1.875	0.75	
S7	2	1.75	0.75	
S8	1.75	2	0.75	
S9	3	0.75	0.75	
S10	0.75	3	0.75	
S11	2	2	0.5	
S12	1	3	0.5	
S13	3	1	0.5	
S14	2.5	1.5	0.5	
S15	1.5	2.5	0.5	

Foils S4, S8 and S13 were used for packing salami slices.

**Table 2:** First impression evaluation.

Film	Adhesivity	Surface	Multiple Bends	Margins Uniformity	Pores/Fissures
S1	high	rough on the outside	yes	yes	-
S2	medium	smooth	yes	yes	-
S3	medium	rough on the outside	yes	yes	-
**S4**	**high**	**very smooth and glossy**	**yes**	**yes**	-
S5	medium	rough on the outside	yes	yes	-
S6	low	rough on the outside	yes	yes	-
S7	medium	rough on the outside	yes	yes	-
**S8**	**medium**	**very smooth**	**yes**	**yes**	-
S9	low	rough, with tendency to tighten	yes	yes	-
S10	medium	smooth	yes	yes	-
S11	medium	very smooth	yes	yes	-
S12	high	smooth	yes	yes, tendency to twist	-
**S13**	**medium**	**very smooth**	**yes**	**yes**	-
S14	low	rough on the outside	yes	yes	-
S15	high	rough on the outside	yes	yes	-

-: represents the absence of pores and/or fissures.

**Table 3:** Characteristics of the analyzed films.

Samples	Drying Time, h	Thickness, µm	Retraction Ratio, %	Water Activity, a_w_	Tensile Strength, MPa	Elongation, %
S1	42	72.30 ^a^ ± 0.83	4.55 ^h^ ± 0.91	0.3686 ^b^ ± 0.15	10.75 ^j^ ± 0.20	22.45 ^k^ ± 0.28
S2	68	55.00 ^c,d^ ±1.01	25.90 ^d,e^ ± 0.87	0.3381 ^c,d^ ± 0.33	16.85 ^g^ ± 0.82	53.10 ^d^ ± 0.27
S3	56	64.20 ^b^ ± 1.48	14.85 ^f^ ± 1.74	0.3970 ^a^ ± 0.66	2.15 ^l^ ± 0.95	17.70 ^m^ ± 0.93
**S4**	**36**	**40.30 ^g,h^ ± 0.96**	**46.50 ^a,b^ ± 0.94**	**0.3321 ^d^ ± 0.33**	**26.75 ^d^ ± 0.11**	**33.30 ^i^ ± 0.21**
S5	72	51.90 ^e,f^ ± 0.83	31.50 ^c,d^ ± 1.10	0.3512 ^c^ ± 0.77	25.45 ^e^ ± 0.19	21.20 ^l^ ± 0.28
S6	56	71.70 ^a^ ± 1.30	5.15 ^g,h^ ± 1.12	0.3300 ^d^ ± 0.15	10.85 ^i,j^ ± 0.19	42.20 ^f^ ± 0.36
S7	62	37.70 ^h,I^ ± 1.51	50.20 ^a^ ± 1.71	0.3131 ^d^ ± 0.72	21.55 ^f^ ± 0.30	42.70 ^e^ ± 0.52
**S8**	**68**	**36.30 ^i^ ± 0.89**	**51.80 ^a^ ± 0.82**	**0.3406 ^c,d^ ± 0.33**	**47.40 ^a^ ± 0.20**	**92.15 ^a^ ± 0.32**
S9	36	66.30 ^b^ ± 1.30	12.45 ^f^ ± 1.32	0.3311 ^d^ ± 0.33	14.45 ^h^ ± 0.37	17.45 ^m^ ± 0.15
S10	72	58.00 ^c^ ± 1.35	22.10 ^e^ ± 1.10	0.3729 ^b^ ± 0.77	2.40 ^l^ ± 0.43	35.35 ^h^ ± 0.30
S11	38	50.30 ^f^ ± 0.91	33.85 ^c^ ± 0.88	0.3136 ^e^ ± 0.15	30.10 ^c^ ± 0.48	72.25 ^b^ ± 0.22
S12	70	53.20 ^d,e^ ± 1.07	29.35 ^c,d^ ± 0.99	0.3375 ^c,d^ ± 0.33	8.95 ^k^ ± 0.22	38.15 ^g^ ± 0.23
**S13**	**36**	**43.50 ^g^ ± 0.67**	**42.40 ^b^ ± 0.59**	**0.3411 ^c,d^ ± 0.25**	**8.55 ^k^ ± 0.33**	**60.85 ^c^ ± 0.19**
S14	42	67.50 ^b^ ± 1.03	10.70 ^f,g^ ± 0.86	0.3254 ^d,e^ ± 0.77	11.25 ^i^ ± 0.14	31.15 ^j^ ± 0.41
S15	60	64.90 ^b^ ± 0.70	14.10 ^f^ ± 0.92	0.3391 ^c,d^ ± 0.33	31.20 ^b^ ± 0.37	42.55 ^e, f^ ± 0.81

Results that do not share the same letter are significantly different (*p* < 0.05).

**Table 4:** Optical properties the materials.

Sample	Color			Transmittance, %	Opacity, A*mm^−1^
	L*	a*	b*		
S1	89.87 ^b,c^ ± 0.36	−5.45 ^b^ ± 0.03	17.56 ^a,b,c^ ± 0.53	16.80 ^i^ ± 0.01	10.15 ^e^ ± 0.11
S2	90.94 ^a,b^ ± 0.37	−5.74 ^e,f,g^ ± 0.02	16.97 ^a,b,c^ ± 0.83	40.70 ^c^ ± 0.71	6.65 ^j^ ± 0.24
S3	90.22 ^a,b^ ± 0.48	−5.47 ^b,c^ ± 0.03	16.33 ^b,c,d^ ± 0.64	19.30 ^h^ ± 0.75	10.25 ^e^ ± 0.32
**S4**	**91.00 ^a^ ± 0.71**	**−5.53 ^c^ ± 0.02**	**14.26 ^e^ ± 0.52**	**70.40 ^a^ ± 0.42**	**2.95 ^l^ ± 0.27**
S5	90.71 ^a,b^ ± 0.04	−5.75 ^f,g^ ± 0.02	17.55 ^a,b,c^ ± 0.11	13.70 ^j^ ± 0.02	17.65 ^c^ ± 0.02
S6	89.95 ^b,c^ ± 0.21	−5.52 ^b,c^ ± 0.01	17.15 ^a,b,c^ ± 0.25	13.68 ^j^ ± 0.05	11.75 ^d^ ± 0.17
S7	90.17 ^a,b,c^ ± 0.11	−5.65 ^d^ ± 0.02	16.88 ^b,c^ ± 0.22	13.81 ^j^ ± 0.06	22.70 ^a^ ± 0.26
**S8**	**90.79 ^a,b^ ± 0.58**	**−5.73 ^e,f,g^ ± 0.02**	**15.01 ^d,e^ ± 0.97**	**68.90 ^b^ ± 0.02**	**3.80 ^k^ ± 0.34**
S9	89.19 ^c^ ± 0.22	−4.98 ^a^ ± 0.04	16.51 ^b, c^ ± 0.19	5.75 ^k^ ± 0.01	18.30 ^b^ ± 0.37
S10	90.42 ^a,b^ ± 0.41	−6.08 ^i^ ± 0.03	17.36 ^a,b,c^ ± 0.93	34.60 ^e^ ± 0.21	6.90 ^h^ ± 0.49
S11	89.83 ^b,c^ ± 0.17	−5.70 ^d,e,f^ ± 0.06	17.77 ^a,b^ ± 0.32	33.60 ^f^ ± 0.03	8.90 ^g^ ± 0.28
S12	89.95 ^b,c^ ± 0.66	−5.99 ^h^ ± 0.02	18.28 ^a^ ± 0.88	39.60 ^d^ ± 0.45	6.90 ^i^ ± 0.30
**S13**	**91.68 ^a,b^ ± 0.89**	**−5.68 ^d,e,f^ ± 0.02**	**17.20 ^a,b,c^ ± 0.51**	**70.50 ^a^ ± 0.23**	**2.95 ^l^ ± 0.15**
S14	90.09 ^a,b,c^ ± 0.38	−5.66 ^d,e^ ± 0.03	16.84 ^b,c^ ± 0.78	22.95 ^g^ ± 0.60	10.10 ^e^ ± 0.72
S15	90.24 ^a,b^ ± 0.25	−5.80 ^g^ ± 0.04	17.11 ^a,b,c^ ± 0.35	23.10 ^g^ ± 0.48	9.50 ^f^ ± 0.49

Results that do not share the same letter are significantly different (*p* < 0.05).

**Table 5:** Pearson correlation.

	Agar (g)	Alginate (g)	Glycerol (g)	Thickness (µm)	Retraction Ratio (%)	Moisture Content (%)	Water Solubility (%)	Transmittance (%)	Opacity (A mm^−1^)
Agar (g)	1								
Alginate (g)	−0.942 **	1							
Glycerol (g)	−0.129	−0.129	1						
Thickness (µm)	−0.016	0.115	0.023	1					
Retraction ratio (%)	0.016	−0.115	−0.023	−1.000 **	1				
Moisture content (%)	−0.018	−0.001	0.561 *	0.323	−0.323	1			
Water solubility (%)	−0.715 **	0.618 *	0.274	−0.108	0.108	0.031	1		
Transmittance (%)	0.164	−0.146	−0.112	−0.698 **	0.698 **	0.128	−0.240	1	
Opacity, Amm^−1^	−0.050	−0.053	0.144	0.243	−0.243	−0.407	0.255	−0.841 **	1

**: Correlation is significant at the 0.01 level (2-tailed). *: Correlation is significant at the 0.05 level (2-tailed).

**Table 6:** Microbiological load (UFC/g) of salami slices after 3 months of storage at refrigeration temperature.

Sample	Total Count	Coliforms	Enterobacteria	*E. coli*	*S. aureus*	*L. monocytogenes*	Yeasts/Molds
Control	83	-	-	2	-	-	9
S4	1	-	-	-	-	-	-
S8	3	-	-	-	1	-	-
S13	15	-	-	-	-	-	-

-: represents the absence of microorganisms on the culture media.

**Table 7:** Total color differences of films after refrigeration time.

Sample	ΔE		
	T_1_—One Week	T_2_—One Month	T_3_—Three Months
Salami kept in conventional packaging material	11.41 ^a^ ± 0.33	10.41 ^a^ ± 0.33	11.57 ^a^ ± 0.42
Salami kept in S4 packaging material	8.56 ^b^ ± 0.67	4.68 ^b^ ± 0.91	11.51 ^b^ ± 0.98
Salami kept in S8 packaging material	7.45 ^c^ ± 0.12	8.59 ^c^ ± 0.12	5.37 ^c^ ± 0.33
Salami kept in S13 packaging material	8.17 ^d^ ± 0.74	8.98 ^d^ ± 0.66	10.07 ^d^ ± 0.17

Results that do not share the same letter are significantly different (*p* < 0.05).

## Data Availability

The data presented in this study are available upon request from the corresponding author.

## References

[1] Asgher M., Qamar S.A., Bilal M., Iqbal H.M. (2020). Bio-based active food packaging materials: Sustainable alternative to conventional petrochemical-based packaging materials. Food Res. Int..

[2] Aykın-Dinçer E., Erbaş M. (2020). Effect of packaging method and storage temperature on quality properties of cold-dried beef slices. LWT.

[3] Bonnet C., Bouamra-Mechemache Z., Réquillart V., Treich N. (2020). Viewpoint: Regulating meat consumption to improve health, the environment and animal welfare. Food Policy.

[4] Hauschild P., Vogel R.F., Hilgarth M. (2021). Influence of the packaging atmosphere and presence of co-contaminants on the growth of photobacteria on chicken meat. Int. J. Food Microbiol..

[5] Xu M.M., Kaur M., Pillidge C.J., Torley P.J. (2021). Evaluation of the potential of protective cultures to extend the microbial shelf-life of chilled lamb meat. Meat Sci..

[6] Pandey V.K., Upadhyay S.N., Niranjan K., Mishra P.K. (2020). Antimicrobial biodegradable chitosan-based composite Nano-layers for food packaging. Int. J. Biol. Macromol..

[7] EUR-Lex-32004R1935-EN-EUR-Lex. https://eur-lex.europa.eu/legal-content/EN/ALL/?uri=celex%3A32004R1935.

[8] Food Contact Materials. https://ec.europa.eu/food/safety/chemical-safety/food-contact-materials_ro.

[9] (2008). Guidelines on submission of a dossier for safety evaluation by the EFSA of a recycling process to produce recycled plastics intended to be used for manufacture of materials and articles in contact with food-Opinion of the Scientific Panel on food additives, flavourings, processing aids and materials in contact with food (AFC). EFSA J..

[10] Matthews C., Moran F., Jaiswal A.K. (2021). A review on European Union’s strategy for plastics in a circular economy and its impact on food safety. J. Clean. Prod..

[11] Horizon 2020 | Horizon 2020. https://ec.europa.eu/programmes/horizon2020/en/home.

[12] Sanches M.A.R., Camelo-Silva C., Carvalho C.D.S., de Mello J.R., Barroso N.G., Barros E.L.D.S., Silva P.P., Pertuzatti P.B. (2020). Active packaging with starch, red cabbage extract and sweet whey: Characterization and application in meat. LWT.

[13] Vinod A., Sanjay M., Suchart S., Jyotishkumar P. (2020). Renewable and sustainable biobased materials: An assessment on biofibers, biofilms, biopolymers and biocomposites. J. Clean. Prod..

[14] Mohamed S.A.A., El-Sakhawy M., El-Sakhawy M.A.M. (2020). Polysaccharides, Protein and Lipid -Based Natural Edible Films in Food Packaging: A Review. Carbohydr. Polym..

[15] Vilarinho F., Stanzione M., Buonocore G., Barbosa-Pereira L., Sendón R., Vaz M., Silva A.S. (2021). Green tea extract and nanocellulose embedded into polylactic acid film: Properties and efficiency on retarding the lipid oxidation of a model fatty food. Food Packag. Shelf Life.

[16] Kumar S., Mukherjee A., Dutta J. (2020). Chitosan based nanocomposite films and coatings: Emerging antimicrobial food packaging alternatives. Trends Food Sci. Technol..

[17] Moeini A., Germann N., Malinconico M., Santagata G. (2021). Formulation of secondary compounds as additives of biopolymer-based food packaging: A review. Trends Food Sci. Technol..

[18] Xiong Y., Li S., Warner R.D., Fang Z. (2020). Effect of oregano essential oil and resveratrol nanoemulsion loaded pectin edible coating on the preservation of pork loin in modified atmosphere packaging. Food Control.

[19] Smaoui S., Ben Hlima H., Tavares L., Ennouri K., Ben Braiek O., Mellouli L., Abdelkafi S., Khaneghah A.M. (2022). Application of essential oils in meat packaging: A systemic review of recent literature. Food Control.

[20] Pérez M.J., Moreno M.A., Martínez-Abad A., Cattaneo F., Zampini C., Isla M.I., López-Rubio A., Fabra M.J. (2020). Interest of black carob extract for the development of active biopolymer films for cheese preservation. Food Hydrocoll..

[21] Jafarzadeh S., Salehabadi A., Nafchi A.M., Oladzadabbasabadi N., Jafari S.M. (2021). Cheese packaging by edible coatings and biodegradable nanocomposites; improvement in shelf life, physicochemical and sensory properties. Trends Food Sci. Technol..

[22] Al-Moghazy M., El-Sayed H.S., Salama H.H., Nada A.A. (2021). Edible packaging coating of encapsulated thyme essential oil in liposomal chitosan emulsions to improve the shelf life of Karish cheese. Food Biosci..

[23] Lima R., Carvalho A., Vieira C., Moreira R., Conte-Junior C. (2021). Green and Healthier Alternatives to Chemical Additives as Cheese Preservative: Natural Antimicrobials in Active Nanopackaging/Coatings. Polymers.

[24] Ahankari S.S., Subhedar A.R., Bhadauria S.S., Dufresne A. (2021). Nanocellulose in food packaging: A review. Carbohydr. Polym..

[25] Ghosh T., Katiyar V. (2021). Edible Food Packaging: An Introduction. Nanotechnology in Edible Food Packaging.

[26] Petkoska A.T., Daniloski D., D’Cunha N.M., Naumovski N., Broach A.T. (2021). Edible packaging: Sustainable solutions and novel trends in food packaging. Food Res. Int..

[27] Verma M.K., Shakya S., Kumar P., Madhavi J., Murugaiyan J., Rao M.V.R. (2021). Trends in packaging material for food products: Historical background, current scenario, and future prospects. J. Food Sci. Technol..

[28] Puscaselu R.G., Besliu I., Gutt G. (2021). Edible Biopolymers-Based Materials for Food Applications—The Eco Alternative to Conventional Synthetic Packaging. Polymers.

[29] Kandeepan G. (2021). Biodegradable Nanocomposite Packaging Films for Meat and Meat Products: A Review. J. Packag. Technol. Res..

[30] Umaraw P., Munekata P.E., Verma A.K., Barba F.J., Singh V., Kumar P., Lorenzo J.M. (2020). Edible films/coating with tailored properties for active packaging of meat, fish and derived products. Trends Food Sci. Technol..

[31] Wang C., Chang T., Dong S., Zhang D., Ma C., Chen S., Li H. (2020). Biopolymer films based on chitosan/potato protein/linseed oil/ZnO NPs to maintain the storage quality of raw meat. Food Chem..

[32] Martiny T.R., Raghavan V., De Moraes C.C., Da Rosa G.S., Dotto G.L. (2020). Bio-Based Active Packaging: Carrageenan Film with Olive Leaf Extract for Lamb Meat Preservation. Foods.

[33] Wongphan P., Harnkarnsujarit N. (2020). Characterization of starch, agar and maltodextrin blends for controlled dissolution of edible films. Int. J. Biol. Macromol..

[34] Olatunji O. (2020). Agar. Aquatic Biopolymers. Springer Series on Polymer and Composite Materials.

[35] Eltabakh M., Kassab H., Badawy W., Abdin M., Abdelhady S. (2021). Active Bio-composite Sodium Alginate/Maltodextrin Packaging Films for Food Containing Azolla pinnata Leaves Extract as Natural Antioxidant. J. Polym. Environ..

[36] Hidayati S., Zulferiyenni, Maulidia U., Satyajaya W., Hadi S. (2021). Effect of glycerol concentration and carboxy methyl cellulose on biodegradable film characteristics of seaweed waste. Heliyon.

[37] Puscaselu R.G., Amariei S., Norocel L., Gutt G. (2020). New Edible Packaging Material with Function in Shelf Life Extension: Applications for the Meat and Cheese Industries. Foods.

[38] ASTM D882-18 Standard Test Method for Tensile Properties of Thin Plastic Sheeting. https://www.astm.org/Standards/D882.

[39] Singh P., Baisthakur P., Yemul O.S. (2020). Synthesis, characterization and application of crosslinked alginate as green packaging material. Heliyon.

[40] Tarique J., Sapuan S.M., Khalina A. (2021). Effect of glycerol plasticizer loading on the physical, mechanical, thermal, and barrier properties of arrowroot (Maranta arundinacea) starch biopolymers. Sci. Rep..

[41] Aitboulahsen M., El Galiou O., Laglaoui A., Bakkali M., Zerrouk M.H. (2020). Effect of plasticizer type and essential oils on mechanical, physicochemical, and antimicrobial characteristics of gelatin, starch, and pectin-based films. J. Food Process. Preserv..

[42] Lim W.S., Ock S.Y., Park G.D., Lee I.W., Lee M.H., Park H.J. (2020). Heat-sealing property of cassava starch film plasticized with glycerol and sorbitol. Food Packag. Shelf Life.

[43] Huntrakul K., Harnkarnsujarit N. (2020). Effects of plasticizers on water sorption and aging stability of whey protein/carboxy methyl cellulose films. J. Food Eng..

[44] Ballesteros-Mártinez L., Pérez-Cervera C., Andrade-Pizarro R. (2020). Effect of glycerol and sorbitol concentrations on mechanical, optical, and barrier properties of sweet potato starch film. NFS J..

[45] Chen J., Wu A., Yang M., Ge Y., Pristijono P., Li J., Xu B., Mi H. (2021). Characterization of sodium alginate-based films incorporated with thymol for fresh-cut apple packaging. Food Control..

[46] Harnkarnsujarit N., Li Y. (2017). Structure-property modification of microcrystalline cellulose film using agar and propylene glycol alginate. J. Appl. Polym. Sci..

[47] Puscaselu R., Gutt G., Amariei S. (2019). Rethinking the Future of Food Packaging: Biobased Edible Films for Powdered Food and Drinks. Molecules.

[48] Fathiraja P., Gopalrajan S., Karunanithi M., Nagarajan M., Obaiah M.C., Durairaj S., Neethirajan N. (2021). Response surface methodology model to optimize concentration of agar, alginate and carrageenan for the improved properties of biopolymer film. Polym. Bull..

[49] Moreira M.D.R., Pereda M., Marcovich N.E., Roura S.I. (2010). Antimicrobial Effectiveness of Bioactive Packaging Materials from Edible Chitosan and Casein Polymers: Assessment on Carrot, Cheese, and Salami. J. Food Sci..

[50] Lourenço S.C., Fraqueza M.J., Fernandes M.H., Moldão-Martins M., Alves V.D. (2020). Application of Edible Alginate Films with Pineapple Peel Active Compounds on Beef Meat Preservation. Antioxidants.

[51] Alexandre S., Vital A.C.P., Mottin C., Prado R.M.D., Ornaghi M.G., Ramos T.R., Guerrero A., Pilau E.J., Prado I.N.D. (2020). Use of alginate edible coating and basil (Ocimum spp) extracts on beef characteristics during storage. J. Food Sci. Technol..

[52] Hosseini M., Jamshidi A., Raeisi M., Azizzadeh M. (2021). Effect of sodium alginate coating containing clove (Syzygium Aromaticum) and lemon verbena ( Aloysia Citriodora ) essential oils and different packaging treatments on shelf life extension of refrigerated chicken breast. J. Food Process. Preserv..

[53] Kang Z.-L., Wang T.-T., Li Y.-P., Li K., Ma H.-J. (2020). Effect of sodium alginate on physical-chemical, protein conformation and sensory of low-fat frankfurters. Meat Sci..

[54] Xavier L.O., Sganzerla W.G., Rosa G.B., da Rosa C.G., Agostinetto L., Veeck A.P.D.L., Bretanha L.C., Micke G.A., Costa M.D., Bertoldi F.C. (2021). Chitosan packaging functionalized with Cinnamodendron dinisii essential oil loaded zein: A proposal for meat conservation. Int. J. Biol. Macromol..

[55] Duran A., Kahve H.I. (2020). The effect of chitosan coating and vacuum packaging on the microbiological and chemical properties of beef. Meat Sci..

